# Expansion of Medicare Coverage for Medical Nutrition Therapy

**DOI:** 10.1001/jamanetworkopen.2025.7716

**Published:** 2025-04-28

**Authors:** Spencer Rowland, Scott Hummel, Geeta Sikand, Eric J. Brandt

**Affiliations:** 1 University of Michigan, Ann Arbor; 2 Veterans Affairs Ann Arbor Health System, Ann Arbor, Michigan; 3 University of California, Irvine College of Medicine, Irvine; 4 Institute for Healthcare Policy and Innovation, University of Michigan, Ann Arbor; 5 Division of Cardiovascular Medicine, Department of Internal Medicine, University of Michigan, Ann Arbor

## Abstract

This cohort study assesses changes in the proportion of Medicare beneficiaries who would qualify for medical nutrition therapy under a proposed coverage expansion.

## Introduction

Medical nutrition therapy (MNT) is provided by a registered dietitian to improve cardiometabolic outcomes, dyslipidemia, hypertension, body mass index, hemoglobin A_1c_, fasting glucose in type 2 diabetes, chronic kidney disease progression, and overall quality of life, leading to health care cost savings.^1,2,3^ Currently, Medicare covers MNT for diabetes, for kidney disease not requiring dialysis, and for up to 36 months after kidney transplantation. The Medical Nutrition Therapy Act of 2023 (hereinafter, MNT act) proposes expanding coverage to include 11 additional conditions.^4^ Effects of the proposal on the quantity of eligible patients are unknown. We aimed to assess the proportion of Medicare beneficiaries in a university health system who would qualify for MNT under this expanded coverage.

## Methods

This cohort study was conducted using deidentified electronic health record (EHR) data and did not involve human participants; therefore, it was exempt from University of Michigan institutional review, and informed consent was waived. We followed the STROBE reporting guideline.

We identified Medicare beneficiaries in the University of Michigan Health System database with an encounter or problem list diagnosis between January 1 and December 31, 2023. Using *ICD-9* or *ICD-10* codes, we identified the 13 conditions in the proposed MNT act. We excluded patients receiving dialysis. We calculated the proportion of patients eligible for MNT under the current and expanded Medicare coverage scenarios. Given that diet is the largest factor associated with early mortality, primarily through its effect on cardiovascular disease (CVD), we calculated the proportion of patients eligible for MNT based on CVD or CVD-specific risk factors (hypertension, dyslipidemia, diabetes, prediabetes, obesity, and kidney disease).^5^ To further evaluate potential changes in eligibility owing to expanded coverage, we also sequentially included conditions by prevalence, starting with the most common and adding in order of increasing frequency. Self-reported race and ethnicity data were derived from EHRs; these data were collected to describe the study population for external validity. Analyses were conducted with Stata, version 16 (StataCorp LLC).

## Results

This study included 143 157 Medicare beneficiaries (mean [SD] age, 70.9 [11.1] years; 53.5% women and 46.5% men). Participants identified as American Indian or Alaska Native (0.4%), Asian (2.3%), Black (7.5%), Native Hawaiian or Other Pacific Islander (0.04%), White (86.1%), or other race or ethnicity (1.7%) (subgroups not explicitly listed); 2.0% refused to answer or data were unknown.

**Figure 1.**
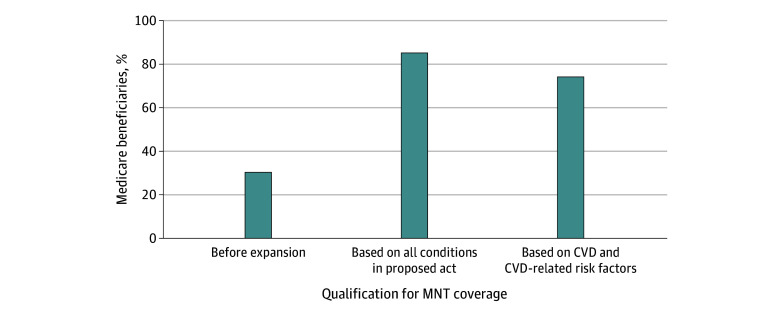
Medicare Beneficiaries Covered Under the Proposed Medical Nutrition Therapy (MNT) Act of 2023 Coverage Expansion Through All Conditions, Cardiovascular Disease (CVD), and CVD-Related Risk Factors

Under current Medicare criteria, 30.3% of beneficiaries were eligible for MNT. However, with the expanded eligibility outlined in the proposed MNT act, 85.1% of all beneficiaries would qualify for MNT. Among all beneficiaries, 74.9% would qualify for MNT based on CVD or CVD-related risk factors, accounting for 88.0% of eligible patients (Figure 1). Only 10.2% would qualify based on other conditions.

## Discussion

A substantial portion of Medicare beneficiaries already qualify for MNT. The findings of this cohort study suggest that the proposed MNT act would make most Medicare participants eligible, primarily due to CVD and CVD-related risk factors. Broadening MNT access has potential to improve management of many chronic health conditions, particularly among those with CVD or CVD-related risk factors. However, a critical factor to consider is the potential strain that expanding MNT could place on health care resources. Increasing the number of registered dietitians may be important to meet expanded demand.

Another key barrier is that although MNT is widely recognized as a cost-effective intervention, expanding coverage to include 11 additional conditions may be perceived by some policy makers as financially unsustainable. Despite strong support from national organizations, the MNT act has repeatedly stalled in Congress, potentially due to concerns regarding costs associated with broad expansion.^6^

**Figure 2.**
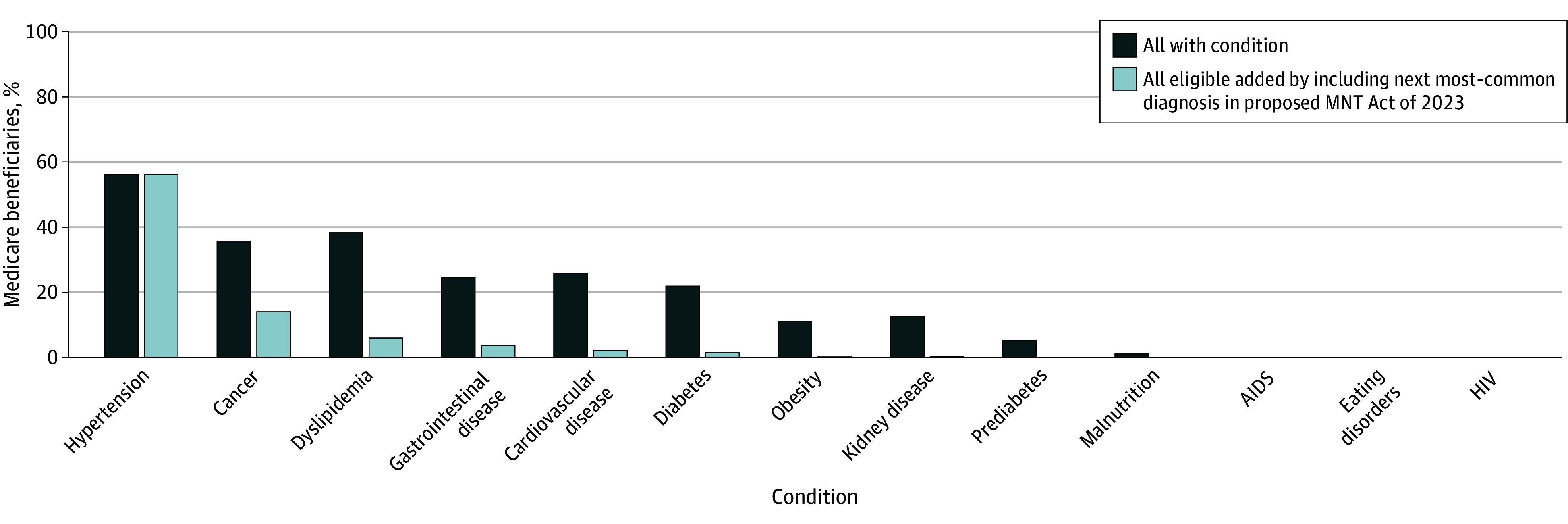
Medicare Beneficiaries With Each Condition, With the Respective Increase in Eligible Participants if the Next Most-Common Diagnosis in the Proposed Medical Nutrition Therapy (MNT) Act of 2023 Is Included

Study limitations include our reliance on EHR data, which may not capture all eligible patients, particularly those with undiagnosed or unrecorded conditions. However, our findings suggest that most potential candidates for MNT coverage could be captured by targeting a smaller set of conditions (Figure 2), primarily CVD or its risk factors. Prioritizing these conditions may present a practical pathway to expansion, balancing feasibility with high-impact chronic disease management and prevention.

## Notes

Supplement.
Data Sharing Statement
