# Supplementary material for: Expansion of Medicare Coverage for Medical Nutrition Therapy
Source: JAMA Netw Open. 2025 Apr 28;8(4):e257716. doi: 10.1001/jamanetworkopen.2025.7716 (PMC12038497; doi:10.1001/jamanetworkopen.2025.7716)
Supplement: Supplement. — Data Sharing Statement [file jamanetwopen-e257716-s001.pdf]

Data Sharing Statement

Rowland. Expansion of Medicare Coverage for Medical Nutrition Therapy. *JAMA Netw Open*. Published April 28, 2025. doi:10.1001/jamanetworkopen.2025.7716

Data
Data available: No

Additional Information
Explanation for why data not available: Data can only be shared in aggregate unless otherwise contracted.
